# Effect of Nutritional Ingredients on Disease Activation in Psoriatic Arthritis

**DOI:** 10.5152/ArchRheumatol.2025.25030

**Published:** 2025-12-01

**Authors:** Gizem Varkal, Neşeriz Nihal Akarca, İpek Türk, Ayşegül Yetişir, Burak Mete

**Affiliations:** 1 Division of Rheumatology, Department of Internal Medicine, Çukurova University Faculty of Medicine, Adana, Türkiye; 2 Department of Nutrition and Dietetics, Çukurova University, Adana, Türkiye; 3 Division of Rheumatology, Department of Physical Medicine and Rehabilitation, Çukurova University Faculty of Medicine, Adana, Türkiye; 4 Division of Public Health, Department of Internal Medicine, Çukurova University Faculty of Medicine, Adana, Türkiye

**Keywords:** Mediterranean diet, obesity, processed foods, psoriatic arthritis

## Abstract

**Background/Aims:**

To investigate the relationship between food composition and patterns and disease activity in psoriatic arthritis (PsA) patients.

**Materials and Methods:**

This cross-sectional study evaluated patients with PsA (n = 73). Sociodemographic and disease-related parameters, including the duration of illness, Disease Activity Index for PsA (DAPSA), C-reactive protein, erythrocyte sedimentation rate (ESR), the Health Assessment Questionnaire (HAQ), and Mediterranean Diet Compatibility Scale were assessed. Nutrient intake was recorded over 3 days and analyzed using a standard nutritional analysis program. Processed food consumption was classified according to the NOVA system.

**Results:**

Among the patients with PsA, 71.2% of patients were female, 42.5% were obese, and 72.6% had low adherence to the Mediterranean diet (MD). The body mass index (BMI) and HAQ scores were higher in those with moderate-severe disease (DAPSA ≥ 15) than in those in remission or with mild disease (DAPSA ≤ 14) (*P* = 0.006 and *P* = 0.008, respectively). The MD adherence score was negatively correlated with the DAPSA, HAQ, and ESR (*P* = .025, *P* = .015, and *P* = .006, respectively). Higher adherence to the MD was associated with a lower risk of moderate-to-severe disease, while increases in cholesterol intake, sodium intake, and BMI were associated with a higher risk of disease severity.

**Conclusion:**

Obesity negatively affects disease progression in PsA. A protein-rich and a low-sodium diet appear to support remission, while poor adherence to the MD and high cholesterol intake may contribute to increased disease activity. These findings emphasize the importance of nutrition in the clinical course of PsA.

**Main Points**

- Nutrition significantly influences the progression of psoriatic arthritis (PsA), similar to its effects on psoriasis and inflammatory bowel disease.
- The Mediterranean diet is recommended for conditions associated with inflammation
- Dietary salt intake has been associated with higher inflammation and disease severity.
- Traditional culinary practices may contribute to reduced disease activity in patients with PsA.

## Introduction

Psoriatic arthritis (PsA) is a complex disease characterized by various clinical symptoms affecting both the musculoskeletal system and the skin. Numerous indices have been introduced to evaluate disease activity in recent years, each considering diverse combinations of illness features.^1^ The Disease Activity Index for PsA (DAPSA) has proven efficacy and reliability for assessing PsA patients’ activity and response to treatment.^2^

Obesity contributes to the pathophysiology of rheumatic disorders, including PsA. Studies have shown that obesity negatively affects the course of these diseases.^3^ Patients with PsA who are obese have lower anti-tumor necrosis factor medication effectiveness.^4^^,^^5^ Elevated body mass index (BMI) has a significant impact on both the pathogenesis and prognosis of PsA.^3^

Diet is a variable risk factor for systemic inflammatory disorders. The Mediterranean diet (MD) is a healthy eating regimen that emphasizes the consumption of abundant amounts of fruits, vegetables, nuts, grains, legumes, fish, shellfish, and extra virgin olive oil.^6^ Research has shown that failure to comply with the MD is a contributing factor in the development of psoriasis and increases the severity of the disease.^7^^,^^8^

Nowadays, the use of processed meals is expanding globally, as ready-made foods constitute a convenience factor. As a result, it is critical to demonstrate the effects of processed foods on health. Food is not healthy or unhealthy simply because it is processed. To properly understand the importance of food processing, a classification is needed that distinguishes between types of processing. The NOVA system classifies foods based on their degree of processing before consumption and is swiftly emerging as a benchmark for food production strategies and industrial food processing. In this system, foods are grouped according to their quality, purpose, and degree of processing. With the identification of ultra-processed foods, the NOVA food classification system is by far the most widely applied system in the scientific literature. The NOVA has categorized all food and food products into 4 groups: group 1 is minimally processed food—unprocessed food, group 2 is processed culinary ingredients, group 3 is processed foods, and group 4 is ultra-processed foods.^9^

Based on the idea that a healthy diet and staying away from ultra-processed foods are factors that directly reduce inflammation, this study aimed to investigate the effect of nutritional content and diet on the disease in PsA patients. The aim of the study was to show the effect of obesity, MD adherence, and dietary content on disease activity.

## Methods

This single-center, cross-sectional study included patients with PsA, identified based on the Classification Criteria for Psoriatic Arthritis (CASPAR) criteria.^10^ Psoriatic arthritis patients who applied to the outpatient rheumatology clinic between December 2023 and March 2024 and consented to take part in the study were enrolled. Patients with cancer, pregnancy, or inadequate cognitive status were excluded from the study. All patients provided informed consent. Sociodemographic and clinical characteristics of the patients were recorded. DAPSA and the Health Assessment Questionnaire (HAQ) were used to assess patients’ levels of activity and health.^2^^,^^11^ A DAPSA score <4 indicates remission; >4 to ≤14, low disease activity; >14 to ≤28, moderate disease activity; and >28.10, high disease activity [2]. The validated HAQ questionnaire was used to assess patient functioning.^12^ Patients were categorized based on HAQ scores as ≥0.5 and <0.5. They were also categorized by BMI for obesity assessment. BMI: <18.4 kg/m^2^ was defined as underweight, 18.5-24.9 kg/m^2^ as normal, 25-30 kg/m^2^ as overweight, 30-44.9 kg/m^2^ as obese, and >45 kg/m^2^ as morbidly obese. The severity of disease activity and acute phase reactants were evaluated, and nutritional status was assessed simultaneously. A qualified dietician assessed the patients’ nutritional status using the MD Compatibility Scale and a 3-day food consumption record. The Nutrition Information System Bebis version 9.0 software was utilized to assess food intake data. Dietary patterns were evaluated using the validated MD Compliance Scale, with scores indicating compliance ≥8 and <8 indicating non-compliance.^13^^,^^14^ The NOVA classification divides foods into 4 categories: unprocessed or minimally processed foods, processed kitchen components, processed foods, and ultra-processed foods.^9^

In addition, to evaluate the nutritional content of PsA patients and healthy individuals, healthy controls of similar age and gender were recruited from health personnel.

The minimum sample size required to be reached was found to be 71 for each group, with a power of 90% CI and a 99% 2-tailed hypothesis test effect size (d) = 0.35 as reference. A total of 144 participants were included: PsA (n = 73) and controls (n = 71).^15^

The study protocol received approval from the Ethics Committee of the Faculty of Medicine at Çukurova University (Date: December 8, 2023, reference number: 139/58). Informed consent was obtained from the patients.

### Statistical Analysis

SPSS version 20.0 (IBM SPSS Corp.; Armonk, NY, USA) software was used for the data analysis. The Shapiro–Wilk test was employed to confirm the normality of the data. The analyses used the Student’s *t*-test, Spearman correlation, and ordinal logistic regression for statistical evaluation. A *P*-value of less than .05 was accepted as statistically significant.

## Results

Seventy-three patients with PsA were included in the study. Table 1 lists the sociodemographic and clinical characteristics of the PsA patients.

No statistically significant difference was detected in MD compliance and nutritional content between the healthy control group and PsA patients. Obesity was substantially higher among PsA patients than in healthy controls (*P* < .001).

Table 2 categorizes the characteristics of moderate-to-severe PsA and mild PsA. When clinical and inflammatory indicators were examined based on the PsA group, the moderate-severe PsA group had significantly higher BMI, C-reactive protein (CRP), erythrocyte sedimentation rate (ESR), and HAQ scores.

When the correlation between the MD adherence score and disease severity and inflammation parameters was examined, it was found that there was a statistically significant negative correlation between the MD adherence score and DAPSA, HAQ, and ESR (Table 3).

In the ordinal logistic regression analysis applied to estimate the risk between disease severity and dietary content, a 1-unit increase in dietary protein reduced the risk of higher disease severity by 0.94 times, a 1-unit increase in iron reduced the risk by 0.64 times. A 1-unit increase in MD adherence reduced the risk by 0.85 times. A 1-unit increase in cholesterol increased the risk of higher disease severity by 1.003 times, a 1-unit increase in sodium by 1.001 times, a 1-unit increase in magnesium by 1.019 times, and a 1-unit increase in BMI by 1.11 times. The effect of nutrient content on disease activity is shown in Table 4.

## Discussion

Only 20% of the PsA had a BMI within the normal range, while the majority were classified as overweight or obese. Obesity is recognized as a contributing factor in the onset of PsA and adversely influences the disease’s progression.^16^ Caso et al^17^ reported a positive connection between DAPSA and BMI in PsA patients. In the current study, individuals with higher disease activity had a higher BMI.

In the study conducted by Caso et al^17^ in PsA patients, they found a negative relationship between DAPSA and compliance with the MD. Additionally, a positive relationship was found between DAPSA and HAQ. Herein, a similar relationship was found between DAPSA and MD compliance. Additionally, a negative correlation was identified between adherence to the MD and both HAQ and ESR. Numerous studies have demonstrated that the MD decreases inflammation. For this reason, the MD is a recommended diet in diseases associated with inflammation.^18^^,^^19^ A study conducted with 61 elderly individuals showed that a high-protein diet and exercise decreased CRP, IL6, and IL10 levels, as well as increased muscle strength.^20^ Protein intake has been found to reduce inflammation in sarcopenic elderly individuals.^21^ In addition to the MD, getting enough protein is especially important in sarcopenic and elderly individuals.

In the current study, dietary salt intake was shown to be a factor that increases inflammation and disease severity. Increased dietary salt consumption has been associated with heightened levels of proinflammatory cytokines in mouse models.^22^ It has been demonstrated in mouse models that a high-salt diet increases the development and severity of autoimmune diseases such as systemic lupus erythematosus.^23^ Yi et al^24^ examined the correlation of monocyte count and inflammatory cytokines with the amount of salt in mice receiving 6-12 g/day. The research indicated a rise in inflammatory cells correlated with elevated dietary salt intake. A study on patients with multiple sclerosis revealed that eating more salt increases the severity of the disease. To the best of knowledge, no one has investigated the correlation between dietary salt consumption and disease activity in patients with PsA. However, it has been shown to be a triggering factor for inflammation.

In the present study, a diet rich in cholesterol was associated with increased severity of PsA disease. A diet high in saturated fats and cholesterol adversely impacts the gut flora and elevates the risk of inflammatory diseases, particularly ulcerative colitis.^25^ Researchers found that the intestinal microbiota became dysbiotic and proinflammatory metabolites increased in a study using mice fed a high-cholesterol diet after cholecystectomy.^26^ The abovementioned data might explain the increase in activity in PsA patients with increased dietary cholesterol.

Traditional methods applied in Indian cuisine have been shown to increase the bioavailability of nutrients and reduce the glycemic index.^27^ Particularly in the traditional culinary culture of the population, the processed culinary components in the current study were non-industrial, seasonal items produced seasonally without the addition of chemicals to increase their shelf life. These include butter, pomegranate juice, olive oil, honey, vinegar, meat-based broths, and sun-dried spices. In Turkish society, traditional culinary methods may have contributed to reduced disease activity in the PsA patients.

The study’s most significant limitations were its small number of patients. The cross-sectional design limited causal inference. Although the results showed that increased salt consumption and cholesterol in the diet increased disease activity, these results cannot be generalized without support from large-sample and prospective studies. In conclusion, a diet high in salt and cholesterol may increase disease activity. Although nutrition has been investigated in the pathogenesis of many diseases, there are few data on this issue in rheumatic diseases. As these data increase, appropriate dietary recommendations for patients may contribute to successful treatment and may help to reduce disease activity. Nutrition is important in the course of PsA disease, as it is in psoriasis and inflammatory bowel disease.

## Figures and Tables

**Table 1.** Sociodemographic and Clinical Characteristics of the PsA Patients

Patients, n	73
Age (years), mean ± SD	51.36 ± 12.18
Female, n (%)	52 (71.2)
Duration of illness, median (min–max)	5 (1-30)
Comorbidities, n (%) Diabetes mellitus Hypertension Coronary artery disease	18 (24.7) 27 (37) 5 (6.8)
DAPSA, n (%) Remission Mild Moderate Severe	12 (16.7) 20 (27.89) 26 (36.1) 14 (19.4)
HAQ score, n (%) ≤0.5 >0.5	51 (76.1) 16 (23.9)
Smoker, n (%)	35 (47.9)
Smoking history (packs-years) median (min–max)	0 (0-60)
BMI (kg/m^2^), n (%) <18.4 18.5-24.9 25-30 30-44.9 >45	2 (2.7) 15 (20.5) 24 (32.9) 31 (42.5) 1 (1.4)
Treatment, n (%) NSAID DMARD Biological treatment ± DMARD	2 (2.7) 38 (52.1) 33 (45.2)
Mediterranean diet compatibility, n (%) Compatible (≥8) Incompatible (<8)	20 (27.4) 53 (72.6)
CRP level (mg/L), median (min–max)	6.8 (1.40-99)
ESR (mm/h), median (min–max)	17.92 ± 11.99

CRP, C-reactive protein; DAPSA, Disease Activity Index for Psoriatic Arthritis; DMARD, Disease-modifying antirheumatic drug; ESR, erythrocyte sedimentation rate; HAQ, Health Assessment Questionnaire; NSAID, non-steroidal antiinflammatory drug.

**Table 2.** Clinical and Inflammatory Parameters, and BMI According to the Severity of PsA

	Mild PsA (DAPSA ≤ 14) (n = 32)	Moderate–Severe PsA (DAPSA > 15) (n = 40)	*P*
Age, years (mean ± SD)	49.56 ± 11.52	53.30 ± 12.30	.192
Duration of illness (mean ± SD)	8.72 ± 7.71	6.70 ± 6.62	.237
Smoking history (packs-years)	6.81 ± 12.98	11.60 ± 15.3	.163
BMI (mean ± SD)	27.90 ± 5.84	31.81 ± 5.86	**.006**
HAQ (mean ± SD)	0.15 ± 0.31	0.43 ± 0.49	**.008**
CRP level (mg/L), mean ± SD	4.43 ± 2.60	15.54 ± 16.62	**.001**
ESR (mm/h), mean ± SD	11.70 ± 7.41	23.10 ± 12.84	**.001**

BMI, body mass index; CRP, C-reactive protein; ESR, erythrocyte sedimentation rate; HAQ, Health Assessment Questionnaire.

**Table 3.** Correlations Between PSA Disease Activation and Mediterranean Diet Adherence

		Mediterranean Diet Compatibility	DAPSA	HAQ	CRP	ESR
Mediterranean diet compatibility	*r *	1.000	−0.232^*^	−0.295^*^	−0.175	−0.330^**^
	*P *		**.025**	**.015**	.147	**.006**
DAPSA	*r *		1.000	0.380^**^	0.715^**^	0.528^**^
	*P *			.002	.000	.000
HAQ	*r *			1.000	0.184	0.213
	*P *				.147	.094
CRP	*r *				1.000	0.550^**^
	*P *					.000
ESR	*r *					1.000
	*P *					

CRP, C-reactive protein; DAPSA, Disease Activity Index for Psoriatic Arthritis; ESR, erythrocyte sedimentation rate; HAQ, Health Assessment Questionnaire.; P, significance; R, Correlation,

**Table 4.** Regression Analysis of Dietary Nutrients Most Associated with Disease Activity in PsA

		Estimate	*P*	95% CI		OR
				Lower Bound	Upper Bound	
Threshold	[DAPSA = 1.00]	−0.235	.867	−2.985	2.515	
	[DAPSA = 2.00]	1.62	.247	−1.124	4.364	
	[DAPSA = 3.00]	3.624	.013	0.75	6.498	
Location	Protein	−0.052	**.005**	−0.089	−-0.016	0.949
	Cholesterol	0.003	**.031**	0	0.006	1.003
	Sodium	0.001	**.049**	2.33	0.001	1.001
	Magnesium	0.019	**.014**	0.004	0.034	1.019
	Iron	−0.434	**.021**	−0.803	−0.065	0.647
	Mediterranean diet compatibility test	−0.162	**.047**	−0.321	−0.002	0.850
	BMI	0.105	**.01**	0.025	0.184	1.110
Link function: Logit						

DAPSA, Disease Activity Index for Psoriatic Arthritis; OR, odds ratio.
